# Exploring the effectiveness of *in ovo* feeding of vitamin C based on the embryonic vitamin C synthesis and absorption in broiler chickens

**DOI:** 10.1186/s40104-021-00607-w

**Published:** 2021-08-03

**Authors:** Yufei Zhu, Jianfei Zhao, Chenxu Wang, Fei Zhang, Xinhuo Huang, Zhouzheng Ren, Xin Yang, Yanli Liu, Xiaojun Yang

**Affiliations:** 1 College of Animal Science and Technology, Northwest A&F University, Yangling, Shaanxi China; 2 Nano Vitamin Engineering Research Center of Shaanxi Province, Xi’an, Shaanxi China

**Keywords:** *In ovo* feeding, L-gulonolactone oxidase, Sodium-dependent vitamin C transporter, Vitamin C, Yolk

## Abstract

**Background:**

Many researches about *in ovo* feeding (IOF) of vitamin C (VC) are gradually carried out to explore physiological development in chicken, but little studies focus on VC synthesis capacity of the embryo itself, the selection of injection site and the effectiveness of IOF of VC. This study aims to explore the above problems.

**Results:**

Kidney and yolk sac were the main organs for VC synthesis and L-gulonolactone oxidase (*GLO*) expression was lower during pre-hatch development than that during post-hatch development. Sodium-dependent vitamin C transporter 1 (*SVCT1*) expression was increased continuously in yolk sac from embryonic age 19 (E19) to post-hatch day 1 (D1) and in intestine (duodenum, jejunum and ileum) from E17 to D1. Plasma VC content was higher at D1 than that at D21 and D42. IOF of VC significantly reduced *GLO* expression in liver, kidney and yolk sac as well as *SVCT1* expression in duodenum, jejunum and ileum, but increased the VC content in plasma, brain, kidney and liver. In addition, IOF of VC obviously reduced the embryonic morality and increased the hatchability under heat stress.

**Conclusions:**

This study suggested that IOF of VC at E11 in yolk was effective for embryonic VC supplementation. These findings provide a theoretical reference about the method of embryonic VC supplementation and effective methodology on embryonic VC nutrition in broiler chickens.

**Supplementary Information:**

The online version contains supplementary material available at 10.1186/s40104-021-00607-w.

## Background

Prenatal nutrition is important for embryonic development and healthy fetus, and even has a persistent consequences from fetus to adult offspring due to the nutri-epigenetics [1, 2]. Unlike mammals, the embryos are separated from the mother during embryonic development in poultry. Eggs are the only source of nutrition. Fortunately, *in ovo* feeding (IOF) technique makes nutrient supplementation possible during embryonic stage [3]. Vitamin C (VC) acts as cofactors of the enzymes related to active DNA demethylation, histone demethylation and other epigenome regulation, possessing a potential to affect epigenetic reprogramming at embryonic stage [4]. Consequently, many researches on IOF of VC are gradually carried out to explore physiological development in chicken, but whether the endogenous VC synthesis during embryonic stage is insufficient, and which injection methods can achieve the best consequence on VC supplementation, whose theoretical evidence is neglected.

For IOF of VC, two questions need to be answered firstly. One is which site is suitable, and another one is what time is suitable. In the previous studies, amniotic fluid and yolk were the main injection sites. The nutrient injected into amniotic fluid can be absorbed by intestine during swallowing process [5, 6], and the nutrient injected into yolk can be absorbed by yolk sac into bloodstream [7]. Based on dynamic development of intestine, changes of amniotic fluid volume and distribution of blood vessel around yolk sac during embryonic stage [8], we speculated that yolk might be a better injection site, because the VC absorption from yolk could start earlier than that from amniotic fluid. Of course, this speculation needs to be investigated. As for injection time, different injection times were selected mainly from embryonic age 11 (E11) to E18 [8–10]. Previously, we had determined the DNA methylation variation trends during embryonic development in broiler chickens [11]. Then, E0, E11 and E15 were selected as potential injection times, and the results of hatchability and growth performance confirmed that E11 was the best choice [12, 13].

Although endogenous VC can be synthesized via the glucuronatexylulose-xylulose cycle in the presence of L-gulonolactone oxidase (*GLO*) [14], it was reported that endogenous synthetic amount was insufficient in previous study [15]. In poultry, liver and kidney were main organs for VC synthesis [14]. Since the yolk sac played part of hepatic function during embryonic stage [16, 17], we speculated that the yolk sac may have the ability to synthesize VC. Sodium-dependent VC transporter 1 (*SVCT1*) and *SVCT2* were responsible for VC absorption and *SVCT1* was the main transporter in endothelial system such as the intestine and yolk sac [18]. Therefore, the mRNA expression of *GLO* and *SVCTs* could reflect respectively VC synthesis and absorption capacity in broilers, but these data are scarce.

The objective of this study aims to clarify whether the VC synthesis capacity is insufficient and whether yolk was suitable as injection site at E11 by analyzing VC synthesis and absorption function during pre-hatch and post-hatch development in broilers. Then, the effectiveness of IOF of VC was evaluated by detecting the VC synthesis and absorption, plasma and tissues VC content as well as VC antioxidant function. Our findings may contribute to provide theoretical reference for the method of embryonic VC supplementation and effective methodology on embryonic VC nutrition in broiler chickens.

## Materials and methods

### Experimental procedures and sample collection

**Fig. 1:**
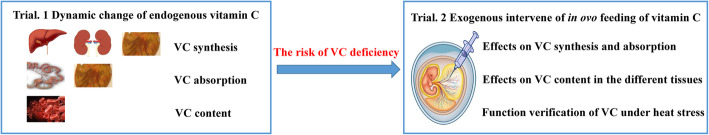
Graphical illustration of the experimental design in this study. *VC*, vitamin C

Graphical illustration of the current study is shown in Fig. 1, and detailed descriptions are as follows:

Trial 1 was conducted to demonstrate whether the endogenous VC synthesis capacity was insufficient and yolk was suitable for the injection site at E11. A total of 200 Arbor Acres breeder eggs were purchased from the Xianyang Dacheng Poultry Industry Co. Ltd. (Xianyang, China). After disinfection, all eggs were randomly divided into eight replicates with 25 eggs per replicate and incubated in an automatic incubator (9TV-2A; Beijing LanTianJiao Electronic Technology Co. Ltd). At E10, all eggs were candled to remove unfertilized eggs and eggs with dead embryos. After hatching, 80 healthy chicks were selected and randomly divided into eight replicates with 10 chicks per replicate. All chicks were raised in a commercial farm and the composition of experimental diet was listed in Table S1 without VC supplementation. At E11, E15, E17, E19, E20, post-hatch day 1 (D1), D21 and D42, one embryo or broiler was selected from each replicate. Before slaughtered, blood sample were collected from jugular vein and plasma samples were obtained by centrifuging at 3,000 r/min for 10 min at 4 °C. Then, yolk sac, liver, kidney and the intestinal mucosa (duodenum, jejunum and ileum) were collected and snap-frozen in liquid N_2_. All samples were stored at − 80 °C for further analysis.

Trial 2 was conducted to explore the effectiveness of IOF of VC. Two incubators were used and named Incubator A and B, where there are three same treatment groups for each incubator including control (CON), normal saline (NS) and VC groups. The 240 eggs were incubated in Incubator A and each group was set eight replicates. The 375 eggs were incubated in Incubator B with 125 eggs for each group. Then, IOF procedure was carried out in both Incubator A and B at E11. Non-injection was were given for CON group; each egg in NS group was injected 0.1 mL sterile normal saline and each egg in VC group was injected 0.1 mL sterile normal saline containing 3 mg vitamin C. Injection method and site were the same as previous publication [13]. At E15, E20 and D1, liver, kidney, yolk sac and the intestinal mucosa (duodenum, jejunum and ileum) were collected in Incubator A. Plasma, brain, lung, heart and hatching muscle of broilers were also collected at D1. At E20, the temperature in Incubator B was set 40 °C for 3 h to record hatchability and embryonic morality under heat stress.

### Quantitative real-time PCR

Total RNA, from yolk sac, liver, kidney and intestinal mucosa (duodenum, jejunum and ileum), was extracted following TRIzol Reagent protocol (AG21102, AG, Changsha, China). The concentration, purity and integrity of RNA samples were verified and cDNA was synthesized with an *Evo M-MLV* RT Kit for qPCR (AG11707, AG, Changsha, China). The mRNA expression of *GLO* in yolk sac, liver and kidney and the mRNA expression of *SVCT1* and *SVCT2* in yolk sac, duodenum, jejunum and ileum were analyzed with a SYBR^®^ Green Premix Pro Taq HS qPCR Kit (AG11701, AG, Changsha, China) on the iCycler IQ5 (Bio-Rad, Hercules, CA, USA). Detailed reaction system was referred to our previous description [13]. The primers are listed in Table S2. All samples were run in triplicate and the average cycle threshold (Ct) values were normalized to β-actin and quantified by the 2 − ^ΔΔCt^ method [19]. Finally, 2 − ^ΔΔCt^ values were normalized to the control group.

### Measurement of vitamin C content in plasma and tissues

The VC content in plasma was measured by a commercial kit (A009, Nanjing Jiancheng Bioengineering Institute, Nanjing, China) according to the manufacturer’s instructions. Tissue samples (brain, kidney, liver, heart, lung and hatching muscle) were washed with pre-cooled saline and 200 mg tissue was used to prepare 10% tissue homogenate. The protein content of 10% tissue homogenate was measured by BCA Protein Concentration Determination Kit (A045, Nanjing Jiancheng Bioengineering Institute, Nanjing, China), while the VC content of 10% tissue homogenate was measured with the same as the detection of plasma. The VC content in plasma and tissues were expressed as μg/mL and μg/mg prot, respectively.

### Embryonic morality and hatchability under heat stress

At E21, newly hatched chicks were firstly considered as alive embryos. For the unshelled eggs, they needed to be opened to check whether there existed the heartbeat using the finger gently on the breast of the embryo. If we can feel the heartbeat, the unshelled eggs were regarded as alive embryos, otherwise as dead embryos. The embryonic morality of fertilized eggs (%) = the number of dead embryos/the number of fertilized eggs × 100; the hatchability of fertilized eggs (%) = the number of newly hatched chicks/the number of fertilized eggs × 100.

### Statistical analysis

The data of embryonic morality and hatchability was analyzed by Chi-square test and the *GLO* expression (liver vs kidney) at D21 and 42 was analyzed by independent sample t-test. And all other data were analyzed by one-way ANOVA using the SPSS 21.0 (SPSS Inc., Chicago, IL, USA). Statistical significance was considered at *P* <  0.05 and trends at *P* <  0.1.

## Results

### Dynamic changes of *GLO* and *SVCT1* expression and plasma vitamin C content

**Fig. 2:**
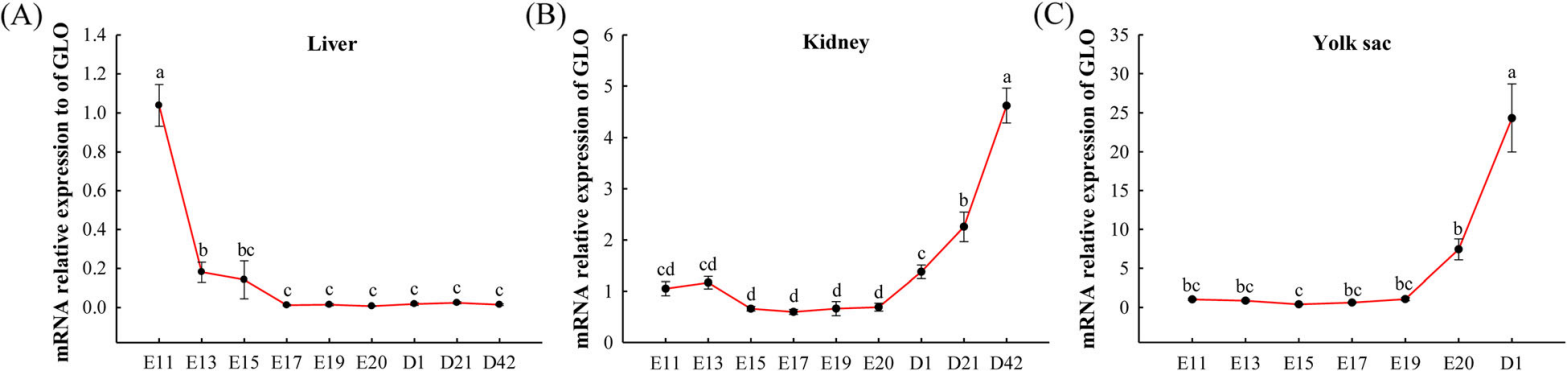
Dynamic *GLO* expression in liver (**A**), kidney (**B**) and yolk sac (**C**) of broilers during pre-hatch and post-hatch development. Values are means ± SEM. ^a–d^ Values with different superscript letters are different (*P* < 0.05, *n* = 8). *GLO*, L-gulonolactone oxidase; E11, embryonic age 11; D1, post-hatch day 1

**Fig. 3:**
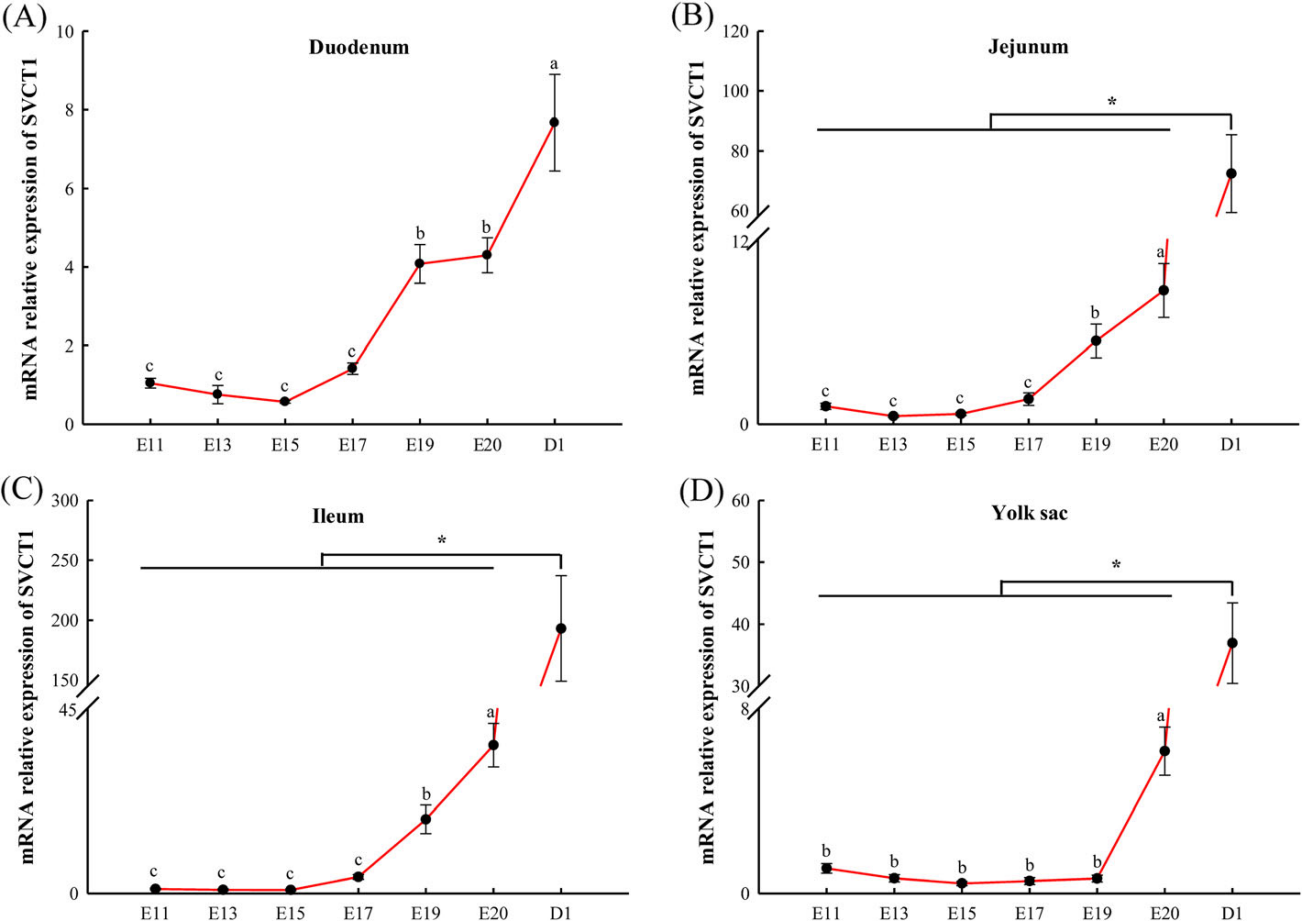
Dynamic *SVCT1* expression in duodenum (**A**), jejunum (**B**), ileum (**C**) and yolk sac (**D**) of broilers during pre-hatch and post-hatch development. Values are means ± SEM. ^a–c^ Values with different superscript letters are different (*P* < 0.05, *n* = 8). *SVCT1*, sodium-dependent vitamin C transporter 1; E11, embryonic age 11; D1, post-hatch day 1

**Fig. 4:**
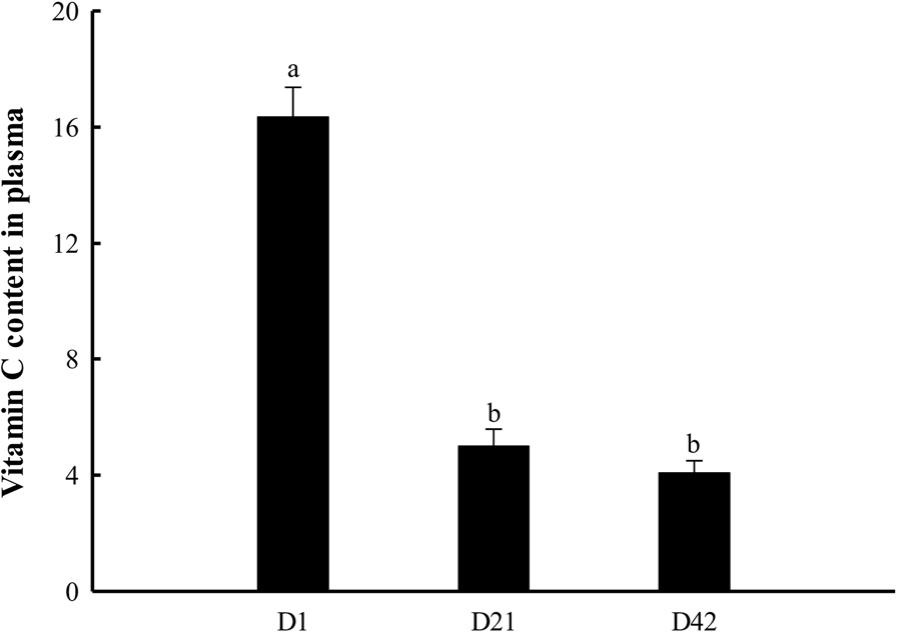
Plasma VC content in broilers at D1, D21 and D42. Values are means ± SEM. ^a–b^ Values with different superscript letters are different (*P* < 0.05, *n* = 8). D1, post-hatch day 1

In liver, *GLO* expression gradually decreased from E11 to E17 and remained steady from E17 to D42 (*P* <  0.05, Fig. 2A). In kidney, GLO expression remained steady from E11 to E20 (*P* > 0.05) and gradually increased from E20 to D42 (*P* <  0.05, Fig. 2B). In yolk sac, *GLO* expression remained steady from E11 to E15 (*P* > 0.05) and gradually increased from E15 to D1 (*P* <  0.05, Fig. 2C). In duodenum, *SVCT1* expression remained steady from E11 to E17 (*P* > 0.05) and gradually increased from E17 to D1 (*P* <  0.05, Fig. 3A). In jejunum, ileum and yolk sac, *SVCT1* expression at D1 was extremely higher than that at embryonic ages (*P* <  0.05, Fig. 3B–D). In jejunum and ileum, *SVCT1* expression remained steady from E11 to E17 (*P* > 0.05) and gradually increased from E17 to D1 (*P* <  0.05, Fig. 3B and C). In yolk sac, *SVCT1* expression remained steady from E11 to E19 (*P* > 0.05) and gradually increased from E19 to D1 (*P* <  0.05, Fig. 3D). As shown in Fig. 4, plasma VC content at D1 was higher than that at D21 and D42 (*P* <  0.05).

### Tissues mRNA expression profile of *GLO* and *SVCT1*

As shown in Table 1, *GLO* expression was higher in kidney than that in liver and yolk sac at E11 and E13, higher in yolk sac than that in liver and kidney at E20 and D1, and lower in liver than that in kidney and yolk sac at E19 and in kidney at D21 and D42 (*P* <  0.05). At E15 and E17, *GLO* expression was highest in kidney and lowest in liver (*P* <  0.05, Table 1). As shown in Table 2, compared with duodenum, jejunum and ileum, *SVCT1* expression was higher in yolk sac at E11, E13, E15, E17, E20 and D1 (*P* <  0.05). At E19, *SVCT1* expression was highest in yolk sac and lowest in duodenum (*P* <  0.05, Table 2).

**Table 1:** Tissues mRNA expression profile of *GLO* among liver, kidney and yolk sac during pre-hatch and post-hatch development in broilers

Ages	Sites			SEM	*P*-value
	Liver	Kidney	Yolk Sac		
E11	1.00^b^	4.81^a^	1.62^b^	0.422	< 0.001
E13	1.00^b^	26.85^a^	7.82^b^	3.000	< 0.001
E15	1.00^c^	60.49^a^	12.46^b^	6.196	< 0.001
E17	1.00^c^	209.76^a^	74.46^b^	20.694	< 0.001
E19	1.00^b^	201.49^a^	140.16^a^	25.510	< 0.001
E20	1.00^b^	471.57^b^	1,737.94^a^	183.759	< 0.001
D1	1.00^b^	334.32^b^	2,019.33^a^	217.332	< 0.001
D21	1.00^b^	441.37^a^	–	56.257	< 0.001
D42	1.00^b^	1,571.28^a^	–	114.289	< 0.001

^a–c^ Means within a row with different superscript letters are different at *P* <  0.05. *GLO*, L-gulonolactone oxidase; E11, embryonic age 11; D1, post-hatch day 1. *n* = 8

**Table 2:** Tissues mRNA expression profile of *SVCT1* among duodenum, jejunum, ileum and yolk sac during pre-hatch and post-hatch development in broilers

Ages	Sites				SEM	*P*-value
	Duodenum	Jejunum	Ileum	Yolk sac		
E11	1.00^b^	2.05^b^	0.84^b^	48.16^a^	3.882	< 0.001
E13	1.00^b^	1.27^b^	0.94^b^	40.24^a^	3.429	< 0.001
E15	1.00^b^	2.15^b^	1.19^b^	35.44^a^	2.868	< 0.001
E17	1.00^b^	2.11^b^	2.26^b^	17.53^a^	1.485	< 0.001
E19	1.00^c^	2.43^bc^	3.53^b^	7.32^a^	0.554	< 0.001
E20	1.00^b^	3.71^b^	6.71^b^	66.53^a^	5.585	< 0.001
D1	1.00^b^	17.38^b^	20.17^b^	280.30^a^	26.087	< 0.001

^a–c^ Means within a row with different superscript letters are different at *P* < 0.05. *SVCT1*, sodium-dependent vitamin C transporter 1; E11, embryonic age 11; D1, post-hatch day 1. *n* = 8

### Effects of *in ovo* feeding of vitamin C on *GLO* expression in different tissues

**Fig. 5:**
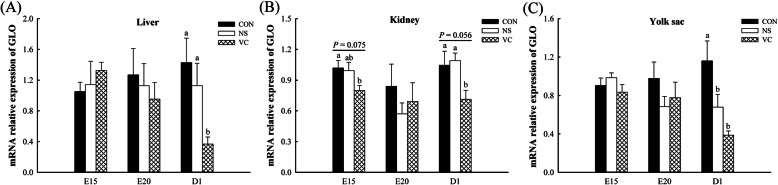
Effects of *in ovo* feeding of vitamin C on the *GLO* expression in liver (**A**), kidney (**B**) and yolk sac (**C**) of broilers. Values are means ± SEM. ^a–b^ Values with different superscript letters are different (*P* < 0.05, *n* = 8). *GLO*, L-gulonolactone oxidase; CON, non-injected control group; NS, normal saline injected group; VC, vitamin C injected group; E15, embryonic age 15; D1, post-hatch day 1

Compared with CON and NS groups, IOF of VC significantly decreased *GLO* expression in liver at D1 (*P* <  0.05, Fig. 5A) and had a decreased trend in kidney at D1 (*P* = 0.056, Fig. 5B). Compared with CON group, *GLO* expression had a decreased trend in kidney at E15 in VC group (*P* = 0.075, Fig. 5B) and was lower in yolk sac at D1 in NS and VC groups (*P* <  0.05, Fig. 5C). And IOF of VC had no significant effects on *GLO* expression in kidney at E20 and in both liver and yolk sac at E15 and E20 (*P* > 0.05, Fig. 5A–C).

### Effects of *in ovo* feeding of vitamin C on *SVCTs* expression in different tissues

**Fig. 6:**
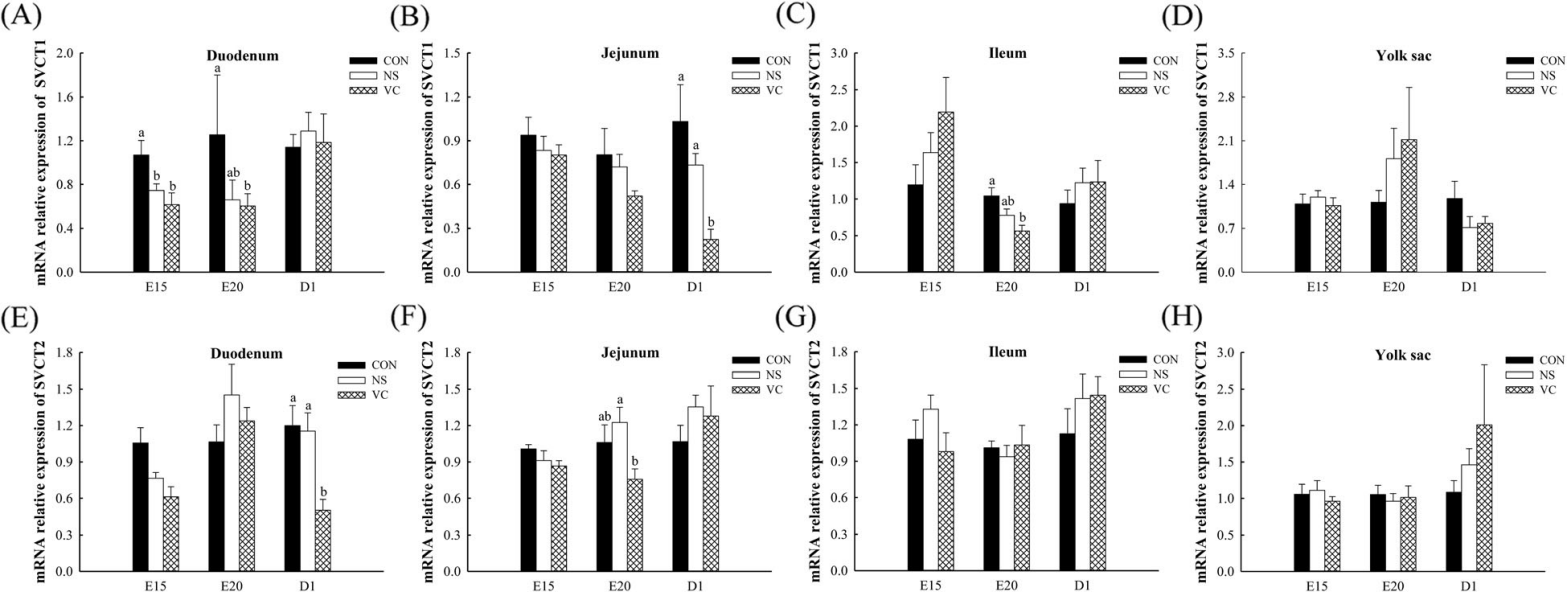
Effects of *in ovo* feeding of vitamin C on the *SVCT1* and *SVCT2* expression in duodenum (**A** and **E**), jejunum (**B** and **F**), ileum (**C** and **G**) and yolk sac (**D** and **H**) of broilers. Values are means ± SEM. ^a-b^ Values with different superscript letters are different (*P* < 0.05, *n* = 8). *SVCT1*, sodium-dependent vitamin C transporter 1; *SVCT2*, sodium-dependent vitamin C transporter 2; CON, non-injected control group; NS, normal saline injected group; VC, vitamin C injected group; E15, embryonic age 15; D1, post-hatch day 1

Compared with CON group, *SVCT1* expression was lower in duodenum at E15 as well as in duodenum and ileum at E20 in VC group (*P* <  0.05, Fig. 6A and C). IOF of VC significantly decreased *SVCT1* expression in jejunum and *SVCT2* expression in duodenum both at D1 (*P* <  0.05, Fig. 6B and E). *SVCT2* expression was down-regulated in jejunum at E20 in VC group (*P* <  0.05, Fig. 6F). And IOF of VC had no significant effects on *SVCT1* or *SVCT2* expression in yolk sac (*P* > 0.05, Fig. 6 D and H) and in intestine at other ages (*P* > 0.05, Fig. 6A, B, C, E, F and G).

### Effects of *in ovo* feeding of vitamin C on plasma and tissues vitamin C content

As shown in Table 3, IOF of VC significantly increased VC content in plasma, brain, kidney and liver (*P* <  0.05), but had no significant effects on VC content in lung, heart and hatching muscle (*P* > 0.05).

**Table 3:** Effects of *in ovo* feeding of vitamin C on plasma and tissue vitamin C content of one-day-old broiler chicks (μg/mg prot)

Items	Treatments			SEM	*P*-value
	CON^1^	NS^2^	VC^3^		
Plasma, μg/mL	18.50^b^	14.62^b^	26.68^a^	1.649	0.003
Brain	6.82^b^	7.94^ab^	10.81^a^	0.737	0.065
Kidney	3.24^b^	3.17^b^	4.45^a^	0.242	0.041
Liver	1.69^b^	1.60^b^	2.51^a^	0.137	0.004
Lung	0.42	0.35	0.32	0.040	0.622
Heart	0.39	0.20	0.33	0.037	0.107
Hatching muscle	0.71	0.89	0.84	0.125	0.855

^a, b^ Means within a row with different superscript letters are different at *P* < 0.05. ^1^ CON, non-injected control group; ^2^ NS, normal saline injected group; ^3^ VC, vitamin C injected group. *n* = 8

### Effects of *in ovo* feeding of vitamin C on embryonic morality and hatchability under heat stress

**Fig. 7:**
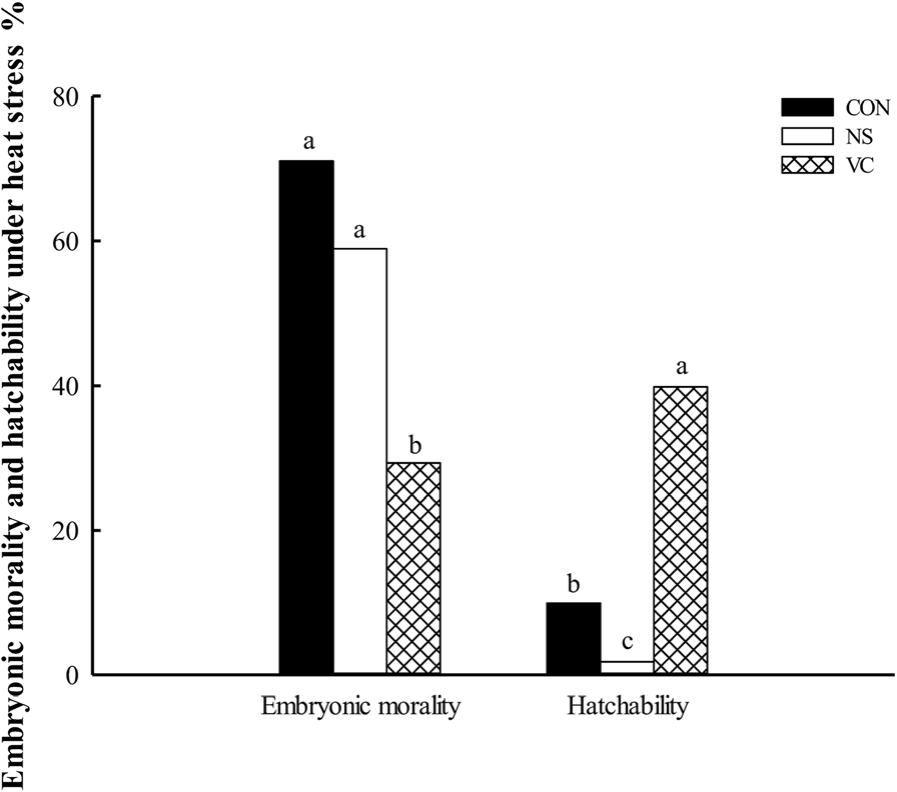
Effects of *in ovo* feeding of vitamin C on embryonic morality and hatchability of fertilized eggs in broilers under heat stress. ^a-b^ Values with different superscript letters are different (*P* < 0.05). CON, non-injected control group; NS, normal saline injected group; VC, vitamin C injected group

As shown in Fig. 7, IOF of VC significantly decreased embryonic morality and hatchability was highest in VC group and lowest in NS group under heat stress (*P* <  0.05).

## Discussion

In poultry, VC could be synthesized by the liver and kidney in the presence of *GLO* [20], but the synthesis capacity in kidney was significantly higher than that in liver [14], which was consistent with our results. In addition, high *GLO* mRNA expression in yolk sac indicated that yolk sac had the ability to synthesize VC. According to tissues mRNA expression profile of *GLO*, kidney and yolk sac were the main sites for VC synthesis during embryonic development, while kidney was the main site for VC synthesis after post-hatch. *GLO* expression of kidney and yolk sac was lower in pre-hatch embryos, suggesting that the capacity of VC synthesis was weak during embryonic stage. However, plasma VC content was higher at D1, which implied that the embryonic VC source may be mainly from endogenous absorption rather than endogenous synthesis. In addition, it was difficult to achieve egg VC deposition by maternal VC supplementation (unpublished data). There may be a risk of VC deficiency during embryonic development.

About the endogenous VC absorption during embryonic development, the part is from the mixture of amniotic fluid and albumen [21, 22], the other is absorbed from yolk sac [17, 23]. Dynamic changes of *SVCT1* expression matched the behavior of embryo swallowing amniotic fluid and yolk sac internalization into abdominal cavity [8, 16]. In other words, the oral consumption of amniotic fluid increased *SVCT1* expression in intestine (duodenum, jejunum and ileum) from E17 to D1 and the internalization of the yolk sac increased *SVCT1* expression from E19 to D1, which together promoted the endogenous VC absorption. Combined with tissues mRNA expression profile of *SVCT1*, yolk sac was the main site for VC absorption from E11 to D1 rather than intestine. Therefore, yolk is more suitable for the injection site than amniotic fluid to reach the purpose of embryonic VC supplementation at E11.

According to our previous studies [11–13], E11 was selected as the injection time based on hatchability and growth performance. And yolk was confirmed as the suitable injection site at E11 in this study. We provided the hypothesis that IOF of VC in yolk at E11 might promote the VC absorption from yolk sac into bloodstream, then increase VC content in tissue and reduce VC synthesis in liver, kidney and yolk sac compensatorily, finally reach biological function of VC in embryos. In order to verify our guess, the trial was carried out to analyze VC synthesis and absorption, the plasma and tissues VC content, and antioxidant function after IOF of VC in yolk at E11.

Dietary VC supplementation significantly reduced the enzyme activity of *GLO* in kidney [14], indicating that VC was absorbed and then reduced endogenous VC synthesis in kidney, which was consistent with our results. However, dietary VC supplementation significantly enhanced the enzyme activity of *GLO* in layer’s liver [14] and reduced that in broiler’s liver [24]. The inconsistent results might be related to poultry breeds [25]. And it was found that IOF of VC reduced *GLO* expression in liver in this study. In addition, the enzyme activity of *GLO* in liver accounted for only 0.5–0.7% compared with that in kidney [14], which represented that the endogenous synthesis capacity was little affected by liver. The yolk sac is a multifunctional organ involved in digestion, transport and metabolism [26]. High *GLO* expression suggested that the glucose contained in yolk could be used as a substrate for VC synthesis in the presence of *GLO* enzyme [17, 27, 28], which together provided the basis for endogenous VC synthesis in yolk sac. In this study, IOF of VC significantly reduced *GLO* expression in liver, kidney and yolk sac, indicating that endogenous VC synthesis was decreased.

The decreased endogenous VC synthesis suggested that IOF of VC may increase endogenous VC absorption. Surprisingly, IOF of VC significantly reduced *SVCT1* and *SVCT2* expression in intestine and had no significant difference on *SVCT1* and *SVCT2* expression in yolk sac. Therefore, high absorption capacity of yolk sac could guarantee the VC absorption even if there was no increasing on *SVCT1* and *SVCT2* expression in yolk sac. On the other hand, the increased VC absorption from yolk could have meet embryos’ needs, which was supported by decreasing *GLO* expression in liver, kidney and yolk sac, and *SVCT1* and *SVCT2* expression in intestine.

VC is widely existing in various tissues, such as brain, kidney, liver, lung, heart and muscle, while VC content shows different distribution [29, 30]. And VC plays an important role in maintaining normal physiological and biochemical functions of tissues [31]. Dietary VC supplementation significantly increased tissue VC content in laying hens [14]. In this study, IOF of VC significantly increased VC content in plasma, brain, kidney and liver, proving that the VC injected in yolk sac has been used by embryos. It was reported that IOF of VC increased the hatchability in our previous study by relieving excessive metabolic heat and reducing the number of dead embryos [32]. Thus, the known biological function of VC was used to verify whether the VC injected could develop function in embryos in this study. The heat stress treatment was performed at E20. Fortunately, IOF of VC significantly reduced embryonic morality and increased hatchability under heat stress in this study, confirming that VC injected exerted the biological function. These results were consistent with the previous study and also proved that the method of IOF of VC was effective for embryonic VC supplementation.

Obviously, there are also some limitations in this study. Blood collection is difficult and VC content in bloodstream had not been detected at embryonic stage. Based on the dynamic changes in intestinal absorption and endogenous synthesis of VC caused by IOF of VC, it was likely that there exists a feedback mechanism, which was needed to be further studied.

## Conclusion

Based on the dynamic changes of *GLO* and *SVCT1* expression and plasma VC content, the risk of VC deficiency was proposed during embryonic development. Yolk sac might be a suitable injection site and E11 was demonstrated as better injection time. VC injected in the yolk could be used by embryo and effectively increased VC content of plasma, brain, kidney and liver, and significantly reduced embryonic morality as well as increased hatchability under heat stress, indicating that IOF of VC at E11 was effective for embryonic VC supplementation. This study provided theoretical reference about the method of embryonic VC supplementation and effective methodology on embryonic VC nutrition in broiler chickens.

## Supplementary Information

**Additional file 1: Table S1.** Composition and nutrient levels of broiler diets on fed basis. **Table S2.** Primer sequence of target genes.

## Data Availability

All data generated or analyzed during this study are available from the corresponding author by request. The datasets supporting the conclusions of this article are included in the article.

## Abbreviations

IOF: *In ovo* feeding
*GLO*: L-gulonolactone oxidase
*SVCT1*: Sodium-dependent vitamin C transporter 1
*SVCT2*: Sodium-dependent vitamin C transporter 2
CON: Control
NS: Normal saline
VC: Vitamin C
