# Supplementary material for: Exploring the effectiveness of in ovo feeding of vitamin C based on the embryonic vitamin C synthesis and absorption in broiler chickens
Source: J Anim Sci Biotechnol. 2021 Aug 3;12:86. doi: 10.1186/s40104-021-00607-w (PMC8330104; doi:10.1186/s40104-021-00607-w)
Supplement: Supplementary file 1 — Additional file 1: Table S1. Composition and nutrient levels of broiler diets on fed basis. Table S2. Primer sequence of target genes. [file 40104_2021_607_MOESM1_ESM.docx]

**Supplementary materials**

**Table S1.** Composition and nutrient levels of broiler diets on fed basis

| Items | Content | |
| --- | --- | --- |
|  | 1-21 d | 22-42 d |
| Ingredient, % |  |  |
| Corn | 59.74 | 60.06 |
| Wheat | — | 5.40 |
| Soybean meal | 25.50 | 18.50 |
| Cottonseed meal | 2.00 | 3.50 |
| Meat and bone meal | — | 2.00 |
| Fish meal | 1.50 | — |
| DDGS^1^ | — | 5.00 |
| Limestone | 1.40 | 0.90 |
| NaCl | 0.33 | 0.35 |
| CaHPO_4_ | 1.50 | 0.60 |
| DL-Met | 0.20 | — |
| L-Lys | — | 0.40 |
| HMA^2^ | 0.20 | 0.13 |
| Thr | 0.03 | 0.05 |
| Middling | 5.00 | — |
| Oil | 1.00 | 1.60 |
| Choline chloride | 0.10 | 0.10 |
| Premix^3^ | 1.50 | 1.50 |
| Total | 100.00 | 100.00 |
| Nutrient levels^4^ |  |  |
| ME^5^, MJ/kg | 12.13 | 12.55 |
| CP^6^ | 20.00 | 18.50 |
| Tatol Lys | 1.17 | 1.00 |
| Tatol Met | 0.49 | 0.40 |
| Tatol Thr | 0.78 | 0.72 |
| Tatol Trp | 0.21 | 0.19 |
| NaCl | 0.33 | 0.35 |
| Ca | 1.00 | 0.90 |
| Total P | 0.66 | 0.60 |
| Available P | 0.42 | 0.38 |

^1^ DDGS means distillers dried grains with soluble; ^2^ HMA means methionine hydroxyl analogue; ^3^ The premix provided the following per kg of diets: VA 9 200 IU, VD_3_ 3 000 IU, VE 38mg, VK_3_ 3 mg, VB_1_ 3 mg, VB_2_ 10 mg, VB_6_ 5 mg, VB_12_ 0.04 mg, niacinaminde 40 mg, calcium pantothenate 16 mg, folic acid 2 mg, biotin 0.3 mg, Fe 66 mg, Cu 15 mg, Mn 95.4 mg, Zn 96.6 mg, I 0.38 mg, Se 0.41 mg; ^4^ Nutrient levels were calculated values; ^5^ ME means metabolizable energy; ^6^ CP means crude protein.

**Table S2.** Primer sequence of target genes

| Gene | Accession  number | Primer  sequences | Product  Size (bp) |
| --- | --- | --- | --- |
| *β-actin* | NM_205518.1 | F: ATTGTCCACCGCAAATGCTTC | 113 |
|  |  | R: AAATAAAGCCATGCCAATCTCGTC |  |
| *SVCT1* | XM_004944768.3 | F: GCTGTACCAGATCGAGGACG | 173 |
|  |  | R: AGGTGAAGATGGTGCCGATG |  |
| *SVCT2* | XM_025142777.1 | F: AGGCAAACACTGGGGTATCG | 247 |
|  |  | R: GCGAGCATAGAAGCCGTACT |  |
| *GLO* | XM_015285218.2 | F: GCCAAGGAGGATTCAAGTT | 167 |
|  |  | R: GATGTCAGAGGGCGAGTG |  |

*SVCT1*, sodium-dependent vitamin C transporter 1; *SVCT2*, sodium-dependent vitamin C transporter 2; *GLO*, L-gulonolactone oxidase.
